# *BDNF* Methylation and Maternal Brain Activity in a Violence-Related Sample

**DOI:** 10.1371/journal.pone.0143427

**Published:** 2015-12-09

**Authors:** Dominik A. Moser, Ariane Paoloni-Giacobino, Ludwig Stenz, Wafae Adouan, Aurélia Manini, Francesca Suardi, Maria I. Cordero, Marylene Vital, Ana Sancho Rossignol, Sandra Rusconi-Serpa, François Ansermet, Alexandre G. Dayer, Daniel S. Schechter

**Affiliations:** 1 Department of Child & Adolescent Psychiatry, University of Geneva Hospitals, Geneva, Switzerland; 2 Department of Psychiatry, Icahn School of Medicine at Mount Sinai, New York, New York, United States of America; 3 Department of Genetic Medicine and Development, Faculty of Medicine, University of Geneva Hospitals, Geneva, Switzerland; 4 Faculty of Health, Psychology and Social care, Manchester Metropolitan University, Manchester, United Kingdom; Nathan Kline Institute and New York University School of Medicine, UNITED STATES

## Abstract

It is known that increased circulating glucocorticoids in the wake of excessive, chronic, repetitive stress increases anxiety and impairs Brain-Derived Neurotrophic Factor (*BDNF)* signaling. Recent studies of *BDNF* gene methylation in relation to maternal care have linked high *BDNF* methylation levels in the blood of adults to lower quality of received maternal care measured via self-report. Yet the specific mechanisms by which these phenomena occur remain to be established. The present study examines the link between methylation of the BDNF gene promoter region and patterns of neural activity that are associated with maternal response to stressful versus non-stressful child stimuli within a sample that includes mothers with interpersonal violence-related PTSD (IPV-PTSD). 46 mothers underwent fMRI. The contrast of neural activity when watching children—including their own—was then correlated to *BDNF* methylation. Consistent with the existing literature, the present study found that maternal *BDNF* methylation was associated with higher levels of maternal anxiety and greater childhood exposure to domestic violence. fMRI results showed a positive correlation of *BDNF* methylation with maternal brain activity in the anterior cingulate (ACC), and ventromedial prefrontal cortex (vmPFC), regions generally credited with a regulatory function toward brain areas that are generating emotions. Furthermore we found a negative correlation of *BDNF* methylation with the activity of the right hippocampus. Since our stimuli focus on stressful parenting conditions, these data suggest that the correlation between vmPFC/ACC activity and *BDNF* methylation may be linked to mothers who are at a disadvantage with respect to emotion regulation when facing stressful parenting situations. Overall, this study provides evidence that epigenetic signatures of stress-related genes can be linked to functional brain regions regulating parenting stress, thus advancing our understanding of mothers at risk for stress-related psychopathology.

## Introduction

Children growing up in households with parents who were victims of violent trauma and who suffer from related psychopathology (i.e. posttraumatic stress disorder [PTSD], major depressive disorder, and borderline personality disorder) may themselves be at elevated risk for developmental psychopathology [1–4]. The literature supports that compromised hypothalamic pituitary adrenal axis (HPA axis) functioning in the wake of excessive, chronic, repetitive stress can be both a marker of and contributing factor to posttraumatic psychopathology [5]. Yet the specific mechanisms by which these phenomena occur remain to be established.

It is known that elevated levels of circulating glucocorticoids induced by chronic stress interferes with *BDNF* signaling and increases anxiety [6]. Both *BDNF* and glucocorticoids regulate the release of Corticotrophin releasing factor in the hypothalamus [7]. Interactions and “cross-talks” likely exist between *BDNF* and *NR3C1* gene expression in stress- related disorders. Maternal prenatal depressive symptoms, for example, predicted both infant *NR3C1* and *BDNF* IV DNA methylation [8].

Researchers have begun to turn their attention to epigenetic factors involved in HPA axis response and adaptation to stressors by studying the methylation status of genes involved in HPA regulation. These genes include the promoter region of *BDNF*. Epigenetic modulation can involve a variety of mechanisms including DNA methylation and can lead to promotion or inhibition of gene-expression. In this paper, we have chosen to focus on *BDNF* methylation, which is the most frequently studied form of epigenetic modulation and which is often associated with an inhibition of gene-expression and protein production.

The animal literature indicates that *BDNF* methylation levels in the brain are modulated by laboratory stressors. One study showed that in rats exposure to prenatal stress decreased *BDNF* mRNA expression and increased *BDNF* methylation in the amygdala and hippocampus among offspring [9]. Furthermore, alterations in *BDNF* methylation status in the hippocampus of Sprague-Dawley rats have been associated with a model for PTSD involving observed avoidance and hyperarousal. These associations were found when the Spague-Dawley rats experienced two acute cat exposures in the context of 31 days of social instability involving daily changing of cage cohorts [10]. Similarly, another study showed that in rats *BDNF* methylation levels are modified by fear conditioning [11] and extinction [12, 13]. In addition, alterations in *BDNF* gene methylation status could be linked to changes in BDNF mRNA levels in the prefrontal cortex during fear conditioning [12].

In humans, methylation of the *BDNF* gene promoter region has also been associated with the development of PTSD, depression, and other trauma-related disorders in children [14]. Similarly, alterations in *BDNF* methylation status have been associated with major depressive disorder [15], suicidal behavior [16, 17] and completed suicide [18] as well as borderline personality disorder and related childhood abuse history [19, 20]. Interestingly, a recent studiy of *BDNF* gene methylation in relation to maternal care have linked high *BDNF* methylation levels in the blood of adults to lower quality of received maternal care measured via self-report [21]. Although the respective study [21] does not allow inference of the causal relationships concerning the pathways involved, it does show that methylation of the *BDNF* gene promoter-region is linked to maternal care and thus may well be a suitable port of entry for the study of familial cycles of maltreatment. Several studies additionally support the notion that caregiver maltreatment history is associated with increased *BDNF* methylation in the amygdala and hippocampus of female rats [22] and that adaptive maternal care is associated with higher *BDNF* protein levels in the brain of the offspring [23, 24].

One way neurotransmitters that are associated with the HPA system could influence the risk for intergenerational transmission of psychopathology is via maternal emotional information processing, and in response, maternal behavior during stressful mother-child interactions. However, little published research has tested this hypothesis. A previous study showed that in a posttraumatically stressed sample, maternal sensitivity was related to activity in brain regions that subserve emotional appraisal, information processing, and regulation (i.e. ventral anterior cingulate cortex; vACC, vmPFC, and orbitofrontal cortex; OFC) when mothers saw emotionally stressful versus non-stressful mother-child interactions [25]. Yet another study showed these regions to be linked to parenting stress in the same sample [26]. Studies of maternal neural activity in response to child stimuli within non-traumatized, non-clinical samples have associated brain regions that subserve emotional information processing with regions that have also been shown to be affected by HPA-axis functioning. In response to exposure to stimuli displaying mothers’ own child‘s distress, several studies have found increased limbic activity (i.e. amygdala and anterior insula) [27–29] as well as the orbitofrontal cortex [30, 31] and anterior cingulate cortex (ACC) in response to parent-child stimuli [32, 33].

The present study examines the link between methylation of the *BDNF* gene promoter region and patterns of neural activity that are associated with maternal response to stressful versus non-stressful child stimuli within a sample consisting of mothers with interpersonal violence-related PTSD (IPV-PTSD) that is above-threshold and sub-threshold for DSM-IV-TR diagnosis as well as non-PTSD control-mothers. To our knowledge no previous study has investigated the relationship of *BDNF* methylation and maternal brain activity using parent-child stimuli in the MRI scanner.

We hypothesized that increased methylation of the *BDNF* gene promoter region would be associated with the following: 1) maternal IPV-PTSD and more generalized anxiety in the context of childhood exposure to IPV, and 2) neural activity of brain regions subserving emotional processing when mothers watched their own and unfamiliar toddlers during a stressful condition (i.e. separation) compared to a non-stressful condition (i.e. free-play).

## Methods

### Participants and Procedures

The institutional ethics committee at the University of Geneva Hospital approved this research project which is in accordance with the Helsinki Declaration [34]. Participants gave written informed consent both for themselves as well as their child. Women and their young children were recruited by flyers posted at the University of Geneva Hospitals and Faculties of Medicine and other Faculty campuses as well as at community centers, daycares, pre-schools, and domestic violence agencies and shelters. All mothers who responded by telephone to the posted flyers and who followed through with an appointment were screened. Fathers and other romantic partners of mothers were not seen in the study given concerns over safety and maintenance of trust for women who had experienced partner violence. Inclusion and exclusion criteria were as follows: biological mothers were included in the study if they had lived with their child for the majority of the child's life since birth, were not experiencing symptoms of psychosis or actively substance abusing and were without mental or physical disability that would preclude participation in research tasks. Due to physiological measurements taken, women who were pregnant or breast-feeding were not accepted into the study. Children were included in the study if they were 12–42 months of age at the time of scheduled mother-child behavioral observations and if they had no mental or physical disability that would preclude participation in research tasks.

Within one month after the screening visit, participants completed two videotaped visits over the ensuing 1–2 month period. During the screening visit, following informed consent, mothers were given a socio-demographic and life-events interview followed by several self-report questionnaires. During the next visit, mothers were interviewed without their child present, with a focus on the mother's mental representations of her child and relationship with her child, an elaboration of her traumatic life-events, followed by structured diagnostic interviews and a series of dimensional measures. Then, 1 to 2 weeks later, mothers were asked to bring their child to the lab for a mother-child interaction procedure otherwise known as the “Modified Crowell Procedure” [35]. This procedure involves free play, separation-reunion, structured play, repeated separation-reunion and exposure to novelty. This mother-child interaction procedure was followed by administration of measures focusing on the child's life events, psychopathology, and social-emotional development. Saliva samples were taken for DNA extraction (as described in more detail below) prior to the Modified Crowell Procedure. After each of these visits, mothers received 50 Swiss francs along with a small book or toy for their child following the parent-child visit.

Mothers who consented and were eligible for MRI scanning, were invited within 2 to 4 weeks after the mother-child visit, to the hospital-based neuroimaging center. After a clinician and neuroimaging specialist-guided orientation to the MRI scanner and scanning process, mothers participated in the fMRI protocol as described below [36].

For a subset of 68 mothers (mean age mothers 34.3 years, SD = 5.8 years, mean age children: 27.2 months, SD. 8.7 months), datasets including successful DNA extraction from saliva were available for *BDNF*. Eight participants were not included in the analysis due to PTSD that was unrelated to violence (accident, medical or traumatic loss). Six additional participants were dropped from the study due to discovery of psychopathology that would exclude them, or having been deemed unreliable informants (i.e. giving discrepant versions of their history). Twenty of the remaining 54 were mothers without PTSD (controls), 9 were subthreshold for IPV-PTSD diagnosis but had clinically significant PTSD symptoms, and 25 mothers were diagnosed with IPV-PTSD. 46 of the participants also successfully completed an MRI scan with data that was analyzable. 20 of those were controls, 8 were subthreshold, and 18 mothers were had an IPV-PTSD diagnosis.

### IPV and other traumatic life events

During the first videotaped interview IPV-exposed and non-IPV-exposed mothers underwent a variety of psychometric evaluations including the Clinician administered PTSD scale (CAPS) [37] to assess lifetime PTSD and the Post-traumatic Symptom Checklist -Short Version (PCL-S) to assess current PTSD symptoms [38]. History of traumatic events during childhood was assessed via the Brief Physical and Sexual Abuse Questionnaire (BPSAQ;[39]), and supplemented with the Traumatic Life Events Questionnaire (TLEQ) [40]. Scoring of the BPSAQ was undertaken as described in a paper by Schechter and colleagues [41]. The severity of physical violence of the mother's partner and herself in the context of adult romantic relationships was measured via the Conflicts Tactics Scale-2, Short Version (CTS2; [42]. Anxiety was measured with the anxiety subscale of the revised version of the Symptom Checklist 90 (SCL-90, [43]). Maternal depressive symptoms were assessed via the Beck Depression Inventory—II [44] as a self-report measure for the current subjective symptom severity.

### Saliva sampling and DNA extraction

Participants were instructed not to eat or drink for one hour prior to the test. Subsequently, a trained technician asked each participant to chew on a Salivette^®^ swab for 3 minutes. The Salivette^®^ swab was then placed in a labeled plastic tube and frozen at -30°C. DNA was extracted with a specific extraction kit (GE Healthcare RPN 8501, Glattbrugg, Switzerland). We conducted quantification analysis of DNA samples with Qubit (the Qubit^®^ 2.0 Fluorometer, Invitrogen) and the quality of DNA fragments was verified with gel electrophoresis. We then modified 2 μg of extracted DNA with sodium bisulfite via EpiTect Bisulfite Kit (Quiagen, Germantown, MD, USA) according to the manufacturer’s protocol. Primers for PCR amplification were performed with HotStarTaq Master Mix Kit (Quiagen, California, USA) on 2 ul of the post bisulfite-treated DNA. The degree of CpG methylation was measured automatically by the Pyro Q-CpG Software (Biotage AB, Uppsala, Sweden). In accordance with a previous study that looked at peripheral vs brain tissue *BDNF* methylation, we used the first 4 CpGs of *BDNF* promoter IV, as they had been shown to be reliable in their assays and to correlate from peripheral measures to hippocampal *BDNF* methylation levels [45].

### MRI procedure

fMRI stimuli were drawn from mother-child interaction sequences of free-play and separation embedded within the 25-minute mother-child interaction (i.e. Modified Crowell Procedure) as described above. A research assistant who was blind to case-control status among mothers' own children selected the silent excerpts for the fMRI stimuli of play and separation: mothers viewed the play-excerpt selected to show the most joy, and reciprocally the separation excerpt that showed the strongest child emotional response in terms of negative emotion and distressed behavior.

Mothers viewed six different silent, 30-second video-excerpts of three children, each during the two conditions (separation and play): 1) own child, 2) unfamiliar boy, and 3) unfamiliar girl. The unfamiliar children conditions were obtained by filming two mothers and their children who did not participate in the study.

The fMRI study design consisted of two runs, each lasting 15 minutes, and each containing 3 blocks during which mothers viewed all six 30-second video excerpts, in a pseudorandom order, counterbalanced within and across runs. Thus, mothers viewed each of the 6 30-second film clips 6 times. Each sequence was preceded by a 2-second white board either saying "mother and child play" or "child during separation". Detailed image acquisition and pre-processing are described in the supporting information. After the MRI visit, mothers received 200 Swiss francs.

### Statistical analyses

Correlations between *BDNF* methylation and behavioral and questionnaire data were performed using the Spearman correlation coefficient. Data were analyzed with SPSS version 22 (IBM Corp., Armonk, NY, USA). Statistical significance was fixed at *p* <0.05. Since we also analyzed CpGs individually, we also report whether significance would survive Bonferroni correction (p-value multiplied by 5, since there are 4 CpGs additional to the overall mean methylation). We, furthermore, performed posthoc tests for each of the clusters that we identified. These posthoc tests correlated the mean activity of each cluster with maternal IPV-PTSD, anxiety and depression symptom severity and *BDNF* methylation levels.

In first level fMRI analysis, we produced a contrast between the average neural activity in response to seeing separation among all children as compared to scenes of play. In 2nd level analysis we applied Pearson correlations to examine the associations between this contrast and mean *BDNF* methylation within a whole-brain analysis. A cluster-extent based thresholding approach was used to correct for multiple comparisons created by the high number of voxels analyzed. A Monte Carlo simulation with 10,000 iterations indicated that a false positive probability of 0.05 was achieved when implementing the condition that each reported regional cluster include at least 27 contiguous voxels (3mm*3mm*3mm) with an uncorrected p < .005. For our whole-brain analysis, the threshold of significance was thus defined as an uncorrected p < .005 with at least 27 contiguous voxels necessary to constitute a significant finding.

## Results

### Correlations of *BDNF* methylation and behavior

**Fig 1:**
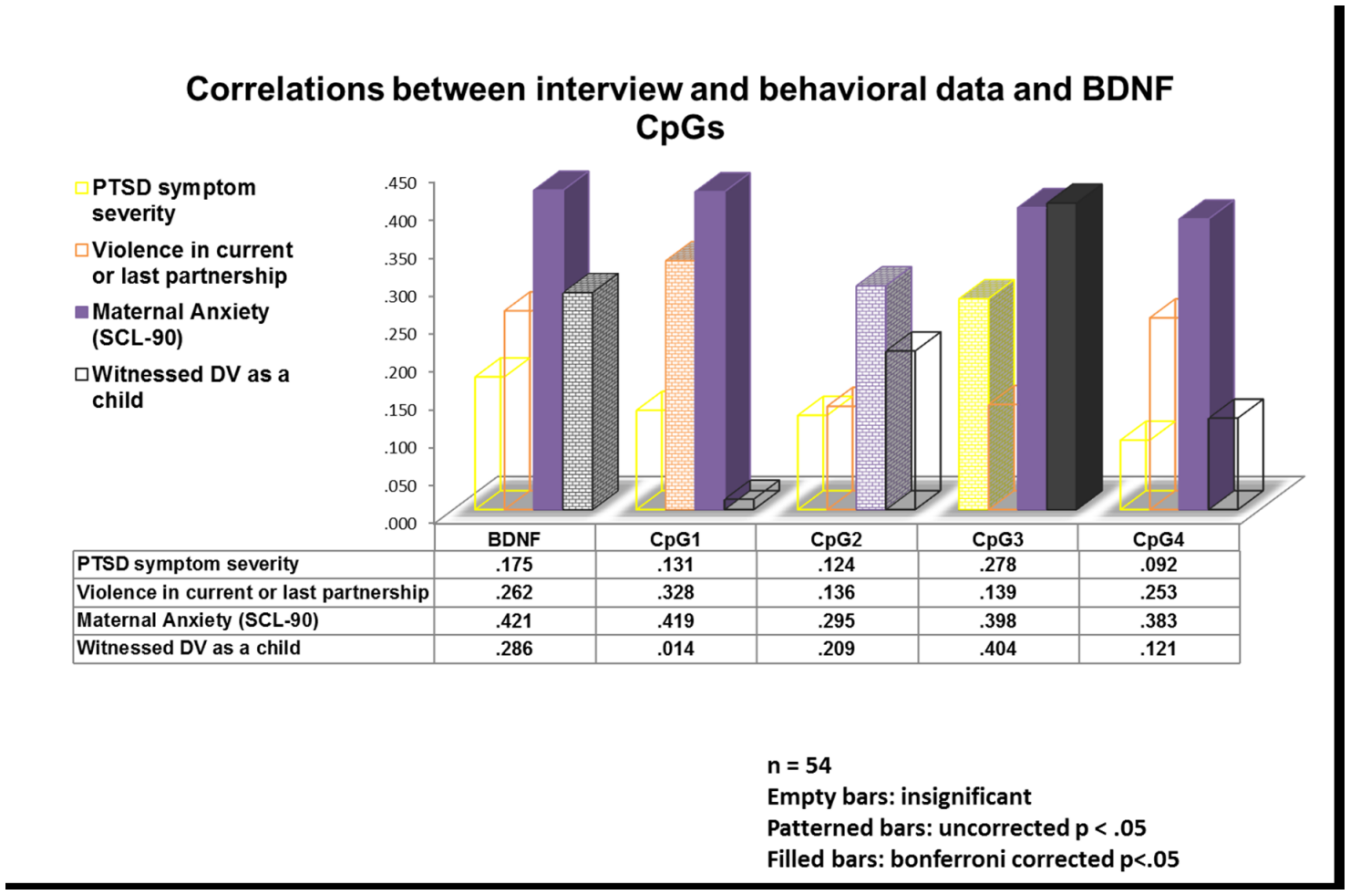
Correlations between IPV-PTSD, violence in the current or last partnership (measured by the conflict tactics scale), anxiety and the experience of domestic violence with the overall mean of BDNF methylation as well as the methylation of individual CpG sites.

Spearman correlations for behavioral data showed that the degree of methylation of exon IV of the *BDNF* gene promoter region as averaged across 4 CpG sites (i.e. mean overall percentage of gene methylation) was positively and significantly correlated with the severity of maternal anxiety (n = 54, r_s_ = .421, p = .002) on the SCL-90. Maternal anxiety severity on the SCL-90 was similarly correlated to the degree of methylation across all CpG sites with the exception of CpG2 which failed to survive the Bonferroni correction, (all n = 54, CpG1: r_s_ = .419, p = .002, CpG 2: r_s_ = .295, p = .030, CpG3: r_s_ = .398, p = .003 CpG4: r_s_ = .383, p = .004). Correlations between *BDNF* methylation and maternal PTSD severity, however, did not remain significant after the Bonferroni correction. There was also no correlation between *BDNF* methylation and maternal depressive symptom severity. Maternal exposure to domestic violence as a child was significantly correlated with the degree of methylation at the CpG3 site (r_s_ = .404, p = .003) even after correcting for Type I error, but was not significantly correlated with the mean overall percentage of *BDNF* methylation across all CpG sites after Bonferroni correction (r_s_ = .286, p = .040; Fig 1).

Maternal IPV-PTSD was correlated to anxiety as measured by the SCL-90 (n = 54, r = .565, p < .001), exposure to domestic violence during childhood (n = 51, r = .414, p = .002), as well as depression (n = 51, r = .624, p < .001). Similarly depression symptoms were also related to anxiety (n = 51, r = .281, p = .046) and exposure to domestic violence as a child (n = 49, r = .305, p = .033). A potential correlation between anxiety and exposure to domestic violence as a child however, failed to reach significance (n = 52, r = .187, p = .184).

### Correlations of BDNF methylation to maternal brain activity

**Fig 2:**
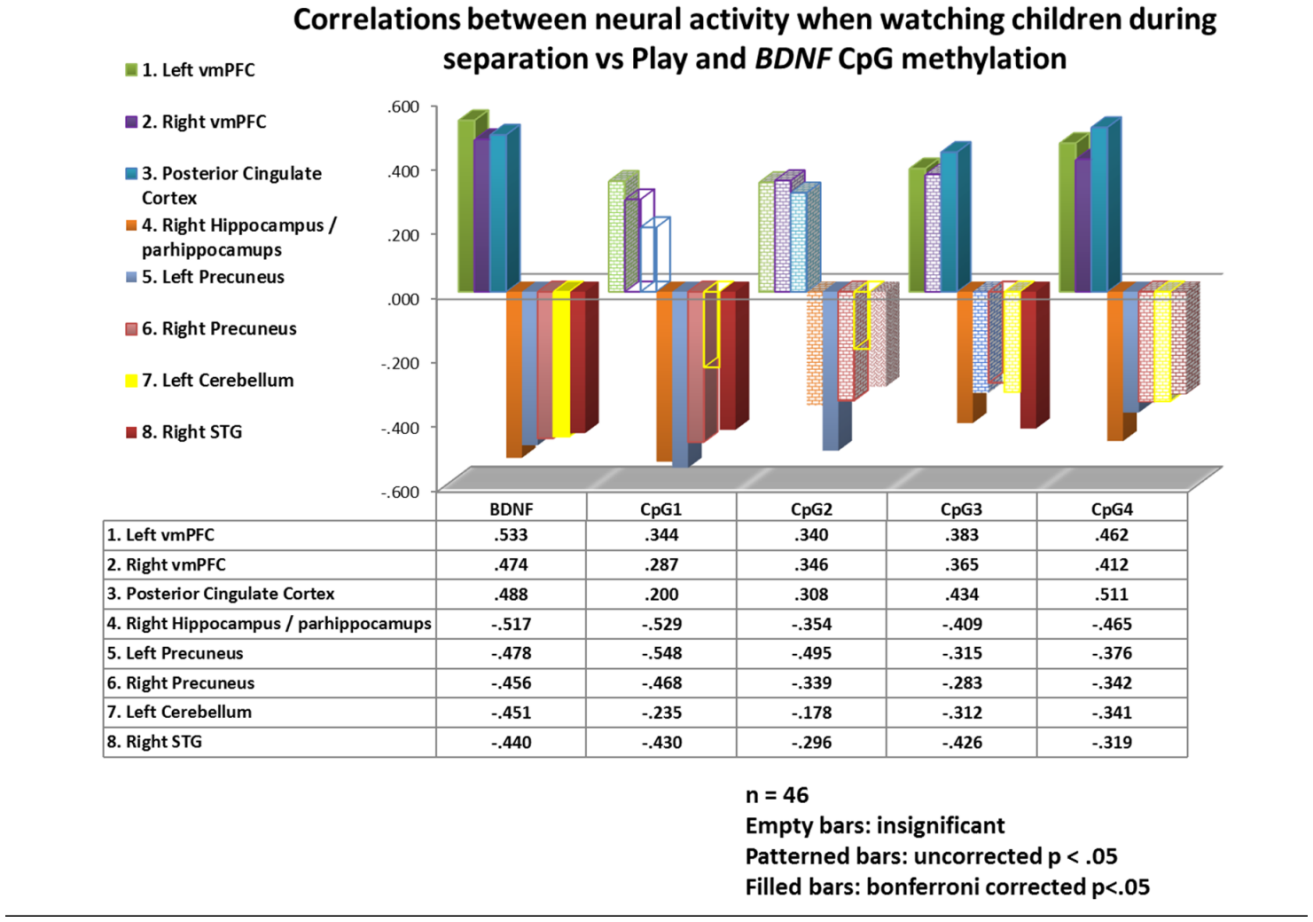
Correlations between neural activity of differing brain regions and the overall mean of *BDNF* methylation as well as the methylation of individual CpG sites.

The contrast of brain activity when mothers watched their own and unfamiliar children during scenes of separation vs play correlated significantly with *BDNF* mean overall methylation in a cluster comprising the left vmPFC, OFC and the vACC (n = 46, r = .533, p < .001), as well as with clusters in the right vmPFC (n = 46, r = .474, p = .001) and posterior cingulate cortex (PCC, n = 46, r = .488, p < .001; Table 1). None of these 3 clusters' activity correlated significantly with IPV-PTSD symptom severity or depression. Post-hoc tests for CpG specific correlations to these clusters can be found in Fig 2.

**Table 1:** Mean percentage of methylation of *BDNF* correlated with BOLD activity when mothers watch separation vs play scenes. Abbreviations: dlPFC = dorsolateral Prefrontal Cortex; dmPFC = dorsomedial Prefrontal Cortex; HC = Healthy Controls; IPV-PTSD = mothers with Interpersonal Violence related Post Traumatic Stress Disorder; OFC = Orbitofrontal Cortex; vmPFC = ventromedial Prefrontal Cortex, r = Pearson correlation, r_s_ = Spearman correlation, p = significance value

	MNI location of the peak voxel				Correlation of cluster activity with *BDNF* rank				
cluster size	x	y	z		r value within:	p value within:	Correlation of cluster activation with rank of *BDNF* mean methylation	Correlation of cluster activation with current PTSD symptom severity	Correlation of cluster activation with current depression symptom severity
					IPV-PTSD	IPV-PTSD			
					Subthreshold	Subthreshold			
				Regions comprised in this cluster	HC	HC			
					.462	.054	r = 0.533	rs = -0.085	rs = -0.007
208	-18	38	-11	Left vmPFC, OFC, sgACC	.641	.087	p < 0.001	p = 0.580	p = 0.960
					.575	.008			
					.475	.046	r = 0.474	rs = -0.060	rs = -0.066
52	15	47	-8	Right vmPFC, OFC	.580	.132	p = 0.001	p = 0.696	p = 0.663
					.412	.071			
					.344	.162	r = 0.488	rs = 0.096	rs = 0.085
37	12	-28	28	Posterior Cingulate Cortex	.573	.138	p < 0.001	p = 0.531	p = 0.575
					.566	.009			
**Negative correlations**									
					-.478	.045	r = -0.517	rs = -0.219	rs = -0.243
217	33	-34	-11	Right Hippocampus, Right Parahippocampal Gyrus, Right Fusiform Gyrus	-.776	.024	p < 0.001	p = 0.149	p = 0.103
					-.410	.073			
					-.600	.009	r = -0.478	rs = -0.151	rs = -0.116
76	-15	-61	64	Left Precuneus	-.349	.396	p = 0.001	p = 0.322	p = 0.443
					-.307	.189			
					-.451	.061	r = -0.456	rs = -0.204	rs = -0.318
37	12	-67	58	Right Precuneus	-.532	.174	p = 0.001	p = 0.178	p = 0.031
					-.311	.182			
					-.569	.014	r = -0.451	rs = -0.154	rs = -0.119
29	-24	-52	-50	Left Cerebellum	.134	.752	p = 0.002	p = 0.314	p = 0.432
					-.553	.011			
					-.525	.025	r = -0.440	rs = -0.022	rs = -0.098
27	48	-37	16	Right Superior Temporal Gyrus	-.688	.059	p = 0.002	p = 0.883	p = 0.516
					-.150	.527			

Mean overall *BDNF* methylation correlated negatively and significantly with maternal neural activity in 5 different clusters: One comprising the right hippocampus and parahippocampus (n = 46, r = -.517, p< .001), one each in the left (n = 46, r = -.478, p = .001) and right precuneus (n = 46, r = -.456, p = .001), one in the left cerebellum (n = 46, r = -.451, p = .002) and one in the right superior temporal gyrus (n = 46, r = -.440, p = .002; Table 1). None of the other clusters correlated with either IPV-PTSD or depression symptom severity. Post-hoc tests for CpG specific correlations to these clusters can be found in Fig 2.

## Discussion

We found, that the severity of maternal anxiety was significantly correlated with mean overall methylation of 4 CpG sites located in exon IV of the *BDNF* promoter region as measured from DNA extracted from mothers’ saliva. In addition, methylation at CpG3 was also significantly associated with maternal exposure to domestic violence during childhood, suggesting that *BDNF* gene methylation levels are modulated by early adverse experiences.

In our study sample, mothers were challenged with an fMRI task during which they visualized their own and unfamiliar toddlers during a stressful condition (i.e. separation) compared to a non-stressful condition (i.e. free-play). We found that increased *BDNF* promoter region methylation was associated to specific patterns of neural activity that subserve psychophysiologic regulation in response to stressful versus non-stressful child stimuli on fMRI. Maternal vmPFC activity (which extended to the OFC and ventral ACC) was associated with *BDNF* methylation. CpG3 and CpG4 sites were the most robustly correlated with neural activity in these regions. These brain regions are generally linked to the processing of competing emotional information, and thus to the regulation of the activity of the amygdala and hippocampus [46, 47]. Patterns of neural activity in these regions may thus regulate maternal behavioral responses to stressful child stimuli, thus impacting the environment of the child. Previous work from our group on individuals examined in this study indicated that maternal neural activity of the vmPFC in response to emotional and potentially stressful adult male-female interactions correlated negatively to observed maternal sensitivity [25].

In addition to the *BDNF gene*, recent work has shown that methylation levels of the glucocorticoid receptor *NR3C1* gene promoter region was correlated with vmPFC activity within this same sample [26]. We interpret the convergence of these findings as supporting the hypothesis that mothers exposed to stressors such as IPV and /or early-life adverse experiences display epigenetic signatures of HPA-axis related genes such as *NR3C1* and *BDNF*. It should be mentioned that *BDNF* methylation levels were correlated to anxiety symptoms and childhood trauma, but not to IPV-PTSD or depression symptoms. In contrast, *NR3C1* methylation levels were negatively correlated to IPV-PTSD [26]. Alterations in the expression of stress-related genes may influence the function of brain networks involved in emotion regulation and affect how mothers perceive and respond to their young children's emotional cues.

Our finding that *BDNF* methylation levels are correlated to hippocampal activity in response to a stressor (child separation) is consistent with animal data indicating that stress affects hippocampal *BDNF* methylation [10]. In addition, *BDNF* deletion in the hippocampus impairs extinction of fear conditioning [48]. A recent study suggested a transgenerational impact on maternal programming by showing that female offspring of traumatized mice had decreased *BDNF* methylation in their hippocampus and an altered reaction to predator-odor [49]. There is additional evidence that *BDNF* methylation is associated with hippocampal activity, fear-conditioned behavior, memory consolidation and retrieval [50]. Given our finding that maternal hippocampal activity in response to the viewing of children during separation is correlated to *BDNF* methylation, future studies should examine the relationship between *BDNF* methylation and mothers’ regulation in the face of displays of negative emotion by their toddlers during routine parent-child interactions. Maternal emotion regulation is known to be linked to the quality of maternal care behavior [25, 51].

## Conclusions

Consistent with the existing literature, the present study found that maternal *BDNF* methylation was associated with maternal anxiety levels and also with childhood exposure to domestic violence [19, 20]. As expected, we found that peripheral *BDNF* methylation is related to a pattern of maternal brain activation that is consistent with corticolimbic dysregulation which we had also previously found to be associated with *NR3C1* methylation [26]. The correlation of vmPFC and OFC activity and *BDNF* methylation levels when mothers watch children during separation vs play suggests that differential *BDNF* methylation and gene expression may occur in these brain regions and impact their function. Since our stimuli focus on stressful parenting conditions, vmPFC activity in these conditions as well as overall *BDNF* methylation may be associated with mothers who are at a disadvantage with respect to emotion regulation when facing stressful parenting situations. The negative correlation with hippocampus activity suggests that such a disadvantage may also have to do with altered emotion generation in response to their children and with the way these emotions are linked to (or deleted from) memory. This study supports the view that early-life adversity may induce long-lasting epigenetic changes in stress-related genes, thus offering clues as to how intergenerational transmission of anxiety and trauma could occur. Finally, further studies are needed in order to understand if psychotherapeutic intervention can modify *BDNF* methylation status and/or neural activity patterns that might also be associated with perceptual and behavioral change that is related to maternal care. Several studies have already demonstrated that change in *BDNF* methylation with intervention is possible [20, 52, 53].

### Limitations

The measures of *BDNF* methylation reported in this paper were taken peripherally. Although previous studies have found that peripheral measures correlate to BDNF methylation in post-mortem human tissue [45], peripheral measures remain an imperfect proxy for central nervous system measures. And thus, their relationship to brain activity must be interpreted with caution. The present study is additionally limited by its associational nature such that we are unable to discern if epigenetic changes might be in some way causing the observed patterns of neural activity, or if the neural activity patterns might be responsible in some way for the maternal epigenetic signatures. As a third option, it may be that another unknown factor is responsible for both. Additionally, this study is limited by a relatively small sample size such that replication of this study within a bigger sample is needed. Finally, this study did not directly associate child behavior or biology to maternal behavior and biology. While this article researches maternal behavior and biology that other studies have linked to child behavior within the same sample, testing for a direct transgenerational link of BDNF methylation to maternal and/or child behavior was not within the scope of this paper.

## Supporting Information

S1 Text Description of the functional MRI data preprocessing. (DOCX)

S1 Data T-value images and their respective headers of the correlation of *BDNF* methylation with the respective in contrast. (ZIP)

S2 Data This is a SPSS database containing the information on of the 54 subjects given in this article with respect to the data presented here. (SAV)
