# Supplementary material for: BDNF Methylation and Maternal Brain Activity in a Violence-Related Sample
Source: PLoS One. 2015 Dec 9;10(12):e0143427. doi: 10.1371/journal.pone.0143427 (PMC4674054; doi:10.1371/journal.pone.0143427)
Supplement: S1 Text — (DOCX) [file pone.0143427.s001.docx]

**Supplementary Materials**

**fMRI Image Acquisition and Pre-Processing**

Scanning took place in with a scanner provided by the Geneva branch of the Center for Biomedical Imaging (CIBM) of the Geneva-Lausanne Universities, the EPFL and the Geneva-Lausanne University Hospitals. All images were acquired on a Siemens 3 Tesla TrioTim scanner using a 12 channel receiver. After a three plane localizing image, a T1-weighted, Spoiled Gradient Recall image was acquired in the sagittal plane to prescribe the location of the anterior commissure–posterior commissure (AC/PC) line. Axial functional images positioned parallel to the AC/PC line were obtained using a T2*-weighted gradient-recalled single-shot echo planar pulse sequence with TR=2100ms, TE=30ms, 80º flip angle, 25.6x25.6cm^2^ field of view, and a 64x64 voxel slice matrix. We acquired 36 slices of 3.5 mm thickness with a spacing of 0.5 mm to provide an effective resolution of 4 x 4 x 3.5 mm^3^. Slices were acquired in interleaved order and spanned the entire brain. The functional echo planar images were preprocessed and statistically analyzed using batch programming based on SPM8 under MATLABR2012a. Prior to analysis, images were visually inspected for major artifacts and signal dropout. Images were realigned to the middle slice of each scan. After motion correction, functional images were co-registered with the anatomical image of the same participant prior to being spatially normalized and reformatted into a 3x3x3 mm resolution space. Finally, normalized images were spatially smoothed using a Gaussian filter with a full-width half-maximum of 8mm.

**fMRI Thresholding**

A cluster-extent based thresholding approach was used to correct for multiple comparisons. A Monte Carlo simulation with 10,000 iterations indicated that a false positive probability of 0.05 was achieved when implementing the condition that a cluster of at least 27 contiguous voxels displays an effect with p<.005.
